# Babesiosis Surveillance, New Jersey, USA, 2006–2011

**DOI:** 10.3201/eid2008.131591

**Published:** 2014-08

**Authors:** Andria Apostolou, Faye Sorhage, Christina Tan

**Affiliations:** Centers for Disease Control and Prevention, Atlanta, Georgia, USA (A. Apostolou);; New Jersey Department of Health, Trenton, New Jersey, USA (A. Apostolou, F. Sorhage, C. Tan)

**Keywords:** babesiosis, surveillance, emerging infections, New Jersey, vector-borne infections, parasites, zoonoses

**To the Editor:** Since zoonotic babesiosis was first identified in the United States in 1966 (*1*), its incidence and geographic range have increased (*2*). Previous studies have demonstrated increases in transfusion-associated cases in recent years (*3*). In 2011, babesiosis became nationally notifiable as its emergence and the potential for transfusion-associated cases were recognized (*2*,*4*). We assessed New Jersey, USA, surveillance data for 2006**–**2011 to characterize case information (incidence, potential transfusion associations, geographic distribution) in a state where babesiosis is endemic.

In New Jersey, babesiosis case reporting began in 1985. A retrospective study identified an upward trend during 1993–2001; eight of 21 counties reported cases (*5*). In 2005, the New Jersey Department of Health established the Communicable Disease Reporting Surveillance System (CDRSS) to collect detailed information for all reportable communicable diseases from clinicians, hospitals, and laboratories. Babesiosis was classified as confirmed for persons who had clinically compatible illnesses and *Babesia* parasites were detected by blood smear examination and as probable for persons who had clinically compatible illness, including documented anemia or thrombocytopenia, and total antibodies, shown by immunoglobulin or IgG titers of >1:256 against *B. microti* by indirect fluorescent test. Cases were considered possibly transfusion associated if patients had documented cellular transfusions with no (or unlikely) other risk factors (e.g., tick bites) reported in CDRSS within 6 months before illness onset. To identify possible transfusion-associated cases, we searched CDRSS text fields for “blood,” “transfusion,” and “receipt of blood donation.” We obtained supportive evidence, when available, for transfusion transmission from medical records or blood center reports. We calculated incidence rates using US Census population data for 2000 (*6*).

During 2006**–**2011, a total of 568 babesiosis cases were reported (Figure); 521 (92%) were classified as confirmed and 47 (8%) as probable. In 2006 and 2011, 64 and 166 cases were reported, a 260% increase in reported cases; respective incidence rates were 0.76 and 1.97 cases per 100,000 population. Seven of New Jersey’s 21 counties accounted for 462 (81%) of all reported cases and for 128 (77%) of the 166 cases occurring during 2011. However, all counties reported at least 1 case within the study period, whereas only 8 counties reported cases during 1993–2001 (*5*) (Technical Appendix Figure). Incidence for 2006**–**2011 ranged from 0.4 to 39.4 cases per 100,000 population; counties in southern New Jersey had the majority of cases and also reported a high incidence of Lyme disease.

**Figure:**
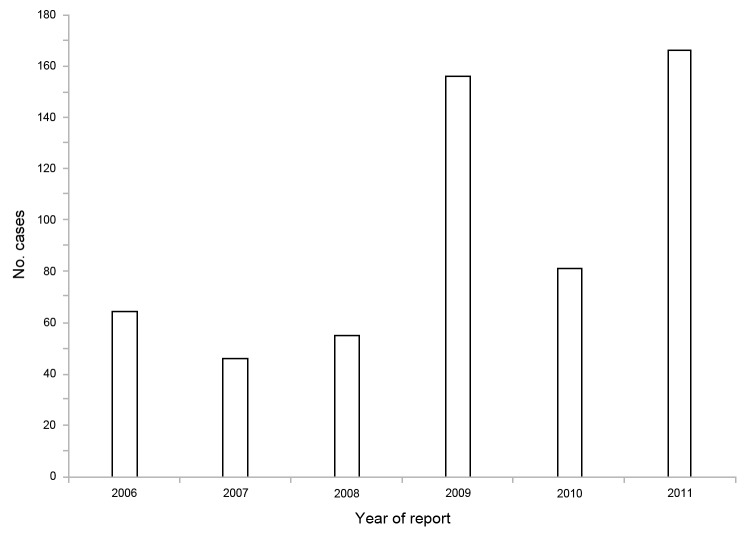
Reported confirmed and probable babesiosis cases, New Jersey, USA, 2006–2011. N = 568.

Case-patients’ median age was 66 years (range 1 month–98 years). Two confirmed cases occurred in infants who were believed to have become infected by congenital transmission (*7*). One infant's mother was asplenic and had confirmed babesiosis. The other mother was asymptomatic and did not meet case criteria but had reported tick bites.

A total of 371 (65%) case-patients were aged >60 years of age; 395 (70%) were male. Of the 568 case patients, 401 (71%) had been hospitalized at least once. Of the 303 case-patients for whom information was available 48 (16%) were admitted to an intensive care unit. The all-cause case-fatality rate was 2% (7/357). All 7 persons who died had been hospitalized, 3 of whom had been admitted to intensive care units.

We identified 12 possible transfusion-associated cases (2 in 2006, 1 in 2007, 3 in 2009, 2 in 2010, and 4 in 2011). Two additional transfusion-associated transmissions (1 each in 2006 and 2009) were identified, but these persons were asymptomatic and not included in this study. Risk factors for possible transfusion-associated cases included surgical procedures with complications requiring transfusions. Median age and case-fatality rate were higher for patients with possible transfusion-associated babesiosis, and these patients were significantly more likely to have acquired infection outside the summer months (Technical Appendix Table).

Our study has some limitations. Increasing awareness, electronic reporting and testing, and environmental or ecologic factors might have contributed to the upward trend and incidence fluctuations. However, neighboring jurisdictions also observed a similar geographic expansion and overall increase in incidence (*8*,*9*). Moreover, New Jersey’s Lyme disease surveillance system shows similar incidence fluctuations for Lyme disease during the study period.

Continued surveillance for detecting babesiosis and investigating possible transfusion-associated cases is needed nationwide (*10*). Although most cases in our study were reported during summer months, possible transfusion-associated cases were reported throughout the year, underscoring the need for constant awareness. The 2 cases of probable congenital infection highlight the need to consider *Babesia* infection for newborns who have compatible clinical manifestations, especially if the mother had risk factors for infection.

Prompt identification of babesiosis is essential to prevent disease transmission from infected blood donors to recipients. Although we modified New Jersey surveillance to include transfusion as a risk factor, collaboration with stakeholders (including blood centers) will further facilitate case detection and confirmation and identification of infected donors. Including babesiosis on the list of nationally notifiable diseases will improve national disease reporting and clarify the geographic distribution and incidence of tickborne and possible transfusion-associated cases. With increasing public awareness and screening, public health professionals and stakeholders might consider dedicating public health resources for babesiosis surveillance.

Technical Appendix Babesiosis incidence rates for 1993–2001 and 2006–2011, New Jersey, USA; and characteristics of tickborne and possible transfusion-associated babesiosis cases, 2006–2011, New Jersey, USA.
