# Supplementary material for: Babesiosis Surveillance, New Jersey, USA, 2006–2011
Source: Emerg Infect Dis. 2014 Aug;20(8):1407–9. doi: 10.3201/eid2008.131591 (PMC4111203; doi:10.3201/eid2008.131591)
Supplement: Technical Appendix — Babesiosis incidence rates for 1993–2001 and 2006–2011, New Jersey, USA; and characteristics of tickborne and possible transfusion-associated babesiosis cases, 2006–2011, New Jersey, USA. [file 13-1591-Techapp-s1.pdf]

Article DOI: http://dx.doi.org/10.3201/eid2008.131591

Babesiosis Surveillance, New Jersey, USA, 2006–2011

Technical Appendix

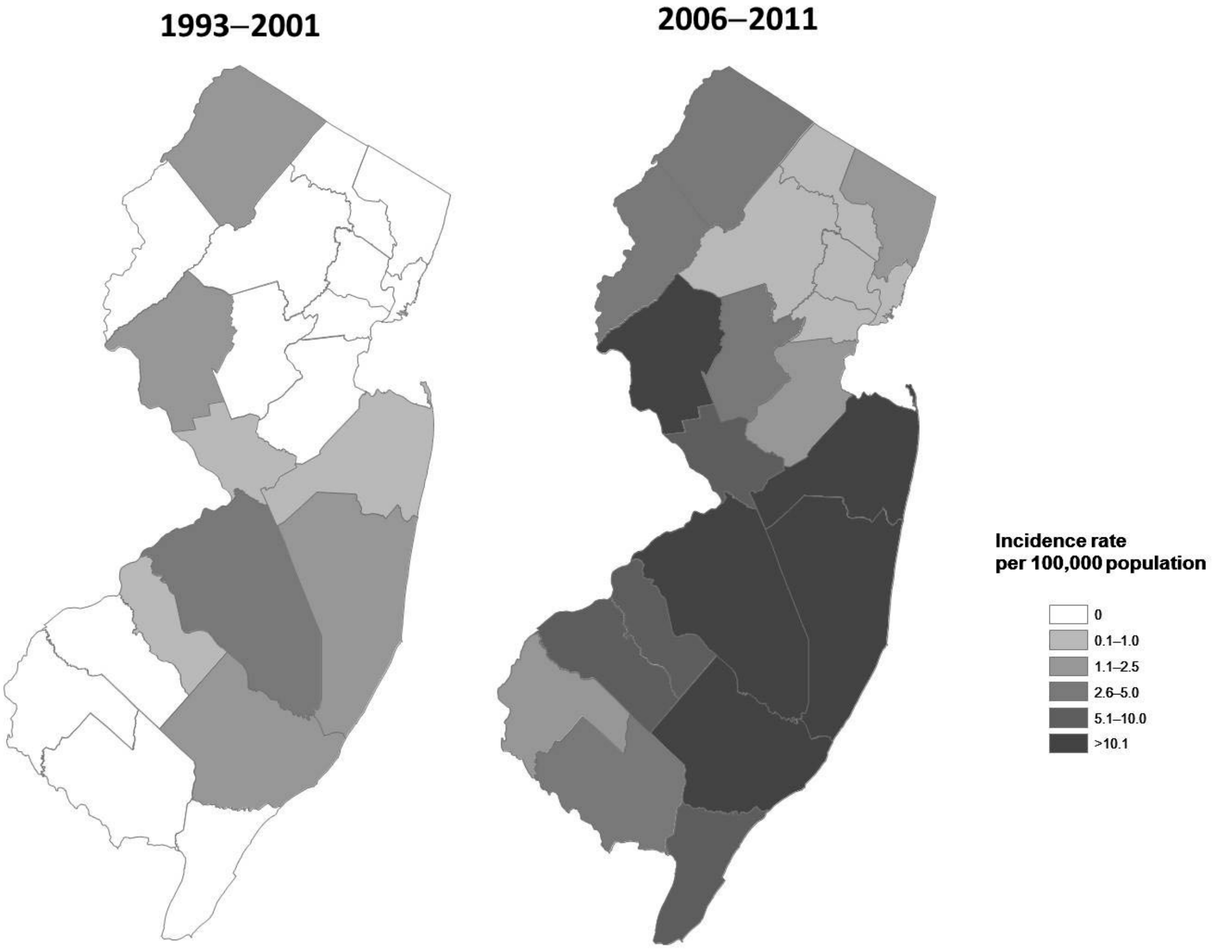

Technical Appendix Figure. Comparison of rates of babesiosis incidence per 100,000 population, New Jersey, USA. Forty cases were reported during 1993–2001 (*1*), and 568 cases were reported during 2006–2011 (this study).

Technical Appendix Table. Characteristics of tickborne and potential transfusion-associated babesiosis cases, New Jersey, USA, 2006–2011

Characteristic	Tickborne cases, n = 556	Possible transfusion-associated cases, n = 12	p value
Median age (range)	66 y (1 mo–98 y)	70 y (39–89 y)	0.5408*
Male sex, no. (%)	388 (70)	7 (58)	0.5262†
June–August illness onset, no. (%)‡	467 (84)	2 (17)	0.0001†§
All-cause mortality, no. (%)¶	6 (1.7)	1 (11)	0.1395†

*2-tailed Wilcoxon rank sum test zval: 0.6116, rank sum: 3.7585e+003.
†2-tailed Fisher exact test.
‡Date of reporting to the New Jersey Department of Health was used when illness onset date was not available.
§p<0.05.
¶Included only case-patients for whom information was available. The denominator for tickborne cases was 348; the denominator for transfusion-associated cases was 9.

Reference

1. Herwaldt BL, McGovern PC, Gerwel MP, Easton RM, MacGregor RR. Endemic babesiosis in another eastern state, New Jersey. Emerg Infect Dis. 2003;9:184–8. PubMed http://dx.doi.org/10.3201/eid0902.020271
